# Metabolomics of Human Semen: A Review of Different Analytical Methods to Unravel Biomarkers for Male Fertility Disorders

**DOI:** 10.3390/ijms23169031

**Published:** 2022-08-12

**Authors:** Janet Blaurock, Sven Baumann, Sonja Grunewald, Jürgen Schiller, Kathrin M. Engel

**Affiliations:** 1 Training Center of the European Academy of Andrology (EAA), Dermatology, Venerology and Allergology Clinic, University Hospital Leipzig, 04103 Leipzig, Germany; 2 Faculty of Medicine, Institute of Legal Medicine, Leipzig University, 04103 Leipzig, Germany; 3 Faculty of Medicine, Institute for Medical Physics and Biophysics, Leipzig University, 04107 Leipzig, Germany

**Keywords:** metabolome, semen, seminal plasma, spermatozoa, male infertility

## Abstract

Background: Human life without sperm is not possible. Therefore, it is alarming that the fertilizing ability of human spermatozoa is continuously decreasing. The reasons for that are widely unknown, but there is hope that metabolomics-based investigations may be able to contribute to overcoming this problem. This review summarizes the attempts made so far. Methods: We will discuss liquid chromatography–mass spectrometry (LC-MS), gas chromatography (GC), infrared (IR) and Raman as well as nuclear magnetic resonance (NMR) spectroscopy. Almost all available studies apply one of these methods. Results: Depending on the methodology used, different compounds can be detected, which is (in combination with sophisticated methods of bioinformatics) helpful to estimate the state of the sperm. Often, but not in all cases, there is a correlation with clinical parameters such as the sperm mobility. Conclusions: LC-MS detects the highest number of metabolites and can be considered as the method of choice. Unfortunately, the reproducibility of some studies is poor, and, thus, further improvements of the study designs are needed to overcome this problem. Additionally, a stronger focus on the biochemical consequences of the altered metabolite concentrations is also required.

## 1. Introduction

Classical semen analysis gathers clinical variables such as sperm concentration, motility and morphology. Laboratories evaluating semen in the light of reproductive health are requested to perform all investigations according to the manual for the examination and processing of human semen edited and constantly updated by the World Health Organization (WHO) to standardize semen analyses worldwide [1]. 

Most studies included in this review article either used the fourth [2] or the fifth edition of the WHO manual for semen analysis [3]. Both editions give reference values that should be fulfilled by an ejaculate to be classified as “normal”. However, reference values have been adjusted and are lower in the fifth compared to the fourth edition. According to the fifth edition, ejaculates should be rated as oligozoospermic if the sperm number is <39 × 10^6^ spermatozoa (fourth ed.: <40 × 10^6^) and/or if the sperm concentration is <15 × 10^6^ cells/mL (fourth ed.: <20 × 10^6^ cells/mL). If there is no sperm detectable, the ejaculate is designated as azoospermic. According to the fourth edition, sperm motility should be classified as (a) rapidly progressive, (b) slowly progressive, (c) nonprogressive but with active tail movement (nonprogressively motile) and (d) immotile without any active tail movement. The fifth edition suggested combining (a) and (b) and ejaculates that contain <40% motile sperm and/or <32% progressively motile sperm are described as asthenozoospermic (AS). However, rapidly progressive spermatozoa have a greater functional potential than slowly progressive ones [4] and, thus, are the sperm cells that most likely reach the oocyte for fertilization.

Morphologically, a spermatozoon is divided into a head, a neck, a midpiece and a tail. These parts, as well as a possible excess residual cytoplasm, are evaluated microscopically after applying a suitable staining method. Morphological evaluations are important because abnormal spermatozoa generally have a lower fertilizing potential [5]. As described in the WHO manuals, a normal human spermatozoon consists of a smooth, oval head (length: 4–5 µm, width: 2.5–3.5 µm) with a definite acrosomal region of 40 to 70% with no more than two small vacuoles (<20% of the head volume) but without any vacuoles in the extra-acrosomal part. The midpiece of a normal human spermatozoon needs to be slender and approximately as long as the head with the same vertical main axis. The tail is thinner than the midpiece, uniform, straight, maybe curved but without any kink, uncoiled and approximately 45 µm in length. The amount of excess residual cytoplasm should be < ⅓ of the head size. These morphological features give information about the functional state of the testicles and the epididymides and, thus, about the process of spermatogenesis. Altered sperm quality in general can also point to infections of the male accessory glands (epididymis, prostate) as well as to disorders on the genetic level. Thus, it is recommended that the category of the observed defects (head, neck and midpiece, tail) is noted. Additionally, any observation of bacteria, immature precursor cells (e.g., spermatides) and immune cells (e.g., leucocytes) within the ejaculate should be annotated. As stated in the fifth edition of the WHO handbook, there should be >4% of morphologically normal sperm in one human ejaculate (14% in the fourth edition). If this is not the case, the ejaculate is classified as teratozoospermic. There are also ejaculates that show more than one abnormality, and are, thus, designated as oligoasthenozoospermic (OA), oligoasthenoteratozoospermic (OAT), asthenoteratozoospermic (AT) or oligoteratozoospermic. In the newest version of the WHO handbook (sixth edition 2021 [6]), it is suggested that the reference limits are abandoned and decision limits “based on clinical and statistical considerations that point to a need for a certain diagnostic or therapeutic intervention” are provided instead.

Despite these microscopically detectable abnormalities, semen analyses often do not give a satisfactory answer in the case of infertility or subfertility because ejaculates are designated as “normal” and the reasons for unwanted childlessness remain unanswered in these cases. Furthermore, as stated in the most recent edition of the WHO manual [6], percentiles do not separate fertile from subfertile men. Therefore, scientists are searching for analytical methods beyond classical semen analyses to find biomarkers that could explain certain (pathological) conditions and/or give hints about potential fertility problems. As early as 2007, Deepinder and colleagues reviewed the role of biomarkers detected via metabolomics (the simultaneous detection of small intermediate or end products of metabolism) for male infertility treatment [7]. In particular, during the past five years, the use of different metabolomic techniques has spurred the search for biomarkers of various infertility causes in human semen. Nevertheless, scientists were searching for characteristic molecules long before the term “metabolomics” arose.

It is also because of the availability of very different techniques that there is no consensus on which biomarker leads to which diagnosis. The selection of the technique as well as details of the sample preparation has an extreme impact on the type of molecules detected in a sample. Whereas only molecules that are present in relatively high absolute amounts (≥100 µg) can be detected by nuclear magnetic resonance (NMR), mass spectrometry (MS)-based techniques also allow for the search of samples with much lower concentrations (typically in the pmol/l and fmol/l range). Furthermore, targeted investigations only look for a limited number of metabolites but with the respective standard substances. As opposed to this, untargeted approaches search for differences in an undefined set of molecules without the possibility of applying internal standards for every compound [8]. Thus, the search for biomarkers for a certain pathological disorder is highly dependent on the methodology used. 

Even though, in the light of abnormal spermiograms, the sperm metabolome is at least as interesting as the seminal plasma (SP), most studies deal with the investigation of SP, probably because SP is much more convenient and easier to obtain. If the seminal plasma is of interest, a human ejaculate can be divided into its liquid (SP) and cellular (sperm) parts by centrifugation [9]. The separation of spermatozoa from the SP without damaging the cells as well as the separation of the sperm cells into different subpopulations (e.g., motile/immotile, mature/immature) can be realized by density gradient centrifugation [10] and, thus, is more challenging. Furthermore, spermatozoa need to be lyzed by a suitable standardized protocol to liberate all the metabolites [11].

This review aims to present an overview of potential biomarkers found for different causes of male infertility. The different compounds will be discussed in relation to the analytical method used for their discovery. The characteristics, advantages and disadvantages of each method mentioned in this review are depicted in Table 1.

## 2. Analytical Methods Used to Study the Human Semen Metabolome

### 2.1. Studies Based on Nuclear Magnetic Resonance Spectroscopy

^1^H NMR offers the possibility of analyzing samples either in an untargeted, and, thus, unbiased manner, or in a targeted approach. However, NMR is not very sensitive and requires (even in the case of ^1^H, the most sensitive nucleus) comparably high concentrations of analytes at least in the µM range [12]. A detailed review on the application of NMR in the field of metabolomics was published only recently by Vignoli and coworkers [13].

While with NMR, analytes can in principle be reliably quantified over a broad concentration range without any suppression effects, most studies aim for a semi-quantitative measurement and, thus, comparison between samples. This tremendously shortens costly measurement time by reducing the relaxation delay. Two additional points emphasize the use of semi-quantification in most studies: (i) for highly abundant water, reliable and easy-to-use water suppression schemes such as excitation sculpting, “watergate” or presaturation are available to tackle this issue [14,15]. However, depending on the scheme used, one has to keep in mind that signals directly under the water resonance may be altered, spectral excitation profile is not uniform anymore and exchangeable groups are also reduced in intensity, respectively. (ii) To suppress other substances which may distort the spectrum with their broad lines, e.g., proteins or other high-molecular-weight substances, a T_2_ filter is normally employed (Carr–Purcell–Meiboom–Gill (CPMG) scheme) or—if these high-molecular-weight substances are of interest—a diffusion-based scheme is used to suppress the intensities of low-molecular-weight substances [16,17].

One additional aspect limits the application of NMR. Biological samples are always aqueous samples on which, for NMR measurements, minimal sample manipulation is performed. Counterintuitively, this may be a drawback, as the spectrum is changing over time after sample preparation due to enzymes [18]. Thus, careful revision of both the preparation and pre-examination should be considered when comparing different studies. Due to the poor sensitivity, studies that used NMR for the analysis of metabolites in semen detected far fewer molecules than those applying mass spectrometry. The results of the following studies are summarized, if not stated otherwise, in Table 2.

In 1993, before the omics era, Hamamah and colleagues published their results on the quantification of glycerylphosphorylcholine (GPC), glycerylphosphorylethanolamine (GPE), citrate and lactate in SP from 21 subjects with an abnormal spermiogram, 14 vasectomized men, 7 OAT patients and 18 normozoospermic controls [19]. These metabolites possessed reasonable intensities and their resonances did not overlap. The concentrations of GPC and lactate were lower in all three groups with semen peculiarities compared to the controls. The amount of lactate directly correlated with the sperm concentration in this study. A few years later, the same group investigated SP from 58 men with nonobstructive azoospermia (azoospermia due to spermatogenic failures) and 17 men with obstructive azoospermia [20]. Healthy controls were not included in this study. For patients with nonobstructive azoospermia, follicle-stimulating hormone (FSH) levels were measured. Depending on the FSH level, there were differences between the patients with nonobstructive azoospermia compared to those with obstructive azoospermia in either the ratios of choline/citrate, choline/lactate and GPC/choline, of choline/lactate and GPC/choline (high FSH) or only in the ratio of GPC/choline (low FSH).

Gupta and coworkers determined the concentrations of ten metabolites, namely the amino acids Ala, Gln, His, Phe and Tyr, as well as choline, citrate, GPC, lactate and uridine, in the SP of 60 fertile, 65 idiopathic and 60 oligozoospermic men using ^1^H NMR on a Bruker Advance 400 MHz spectrometer [21]. For quantification purposes, they used sodium-3-trimethylsilyl-[2,2,3,3-d_4_]-propionate (TSP) as the standard substance because this compound is—in contrast to established tetramethylsilane (TMS)—soluble in water. TSP is a widely used reference compound, although it was shown some time ago that it binds to proteins. In this way, the TSP resonance is severely broadened and the concentration of the standard is, thus, underestimated [22]. This is a particular problem if samples rich in proteins are to be analyzed, but it can be overcome by using different standards, a filtration step or a coaxial insert for the internal standard. In the aforementioned study, Gupta et al. reported that the concentrations of citrate and GPC were significantly reduced in idiopathic and oligozoospermic patients; the concentration of Phe, however, was increased. The authors further reported that other amino acids, such as Val, Leu, Ile, Arg, Glu and Lys, could not be quantified due to overlaps with other resonances. This problem could have been overcome by using 2D NMR, but this (i) would have taken much more time and (ii) quantitative data analysis would have been more difficult. Among the clinical parameters, lipid peroxides (LPOs, products of lipid peroxidation) were significantly higher in both patient groups compared to controls. Unfortunately, the authors do not provide an explanation for the LPO determination.

A study comprising 103 men looked for markers from SP indicating different causes of infertility vs. 6 control samples from men who recently fathered a child [23]. The scientists used a 700 MHz device from Bruker. Samples were diluted in D_2_O-containing 4,4-dimethyl-4-silapentane-1-sulfonic acid (DSS-d_6_) as a reference and quantification standard, and high-resolution ^1^H NMR spectra were recorded. Interestingly, the authors suggested a metabolic reason for idiopathic infertility. Compared to the control group, idiopathic samples (*n* = 17) were lower in valine, lysine, 2-hydroxyisovalerate, hippurate and fructose. As expected, fructose levels were also lower in azoospermic (*n* = 20) and oligozoospermic patients (*n* = 20) compared to controls. It has to be noted that the authors reported percentages of motile sperm and normal morphology for AS (*n* = 20; 43% motile) and teratozoospermia (*n* = 20; 17% morphologically normal), respectively, that are considered as normal according to the WHO guidelines from 2010 [3], which were already valid back then. This might be one reason why the “comparison of the control group with either AS or teratozoospermia failed to reveal any significant difference”. Another reason might be the insensitivity of NMR and the very limited number of unequivocally assigned molecules that ignores the vast majority of (non-NMR-detectable) metabolites. 

In 2015, Zhang and coworkers investigated semen of 30 AS men and 33 proven fathers by ^1^H NMR on a Bruker Avance 600 MHz spectrometer with TSP as internal concentration standard [24]. Unfortunately, information on the separation of sperm and SP is missing in this paper. The authors showed a significant lower lipid concentration in AS men compared to healthy controls and state that this difference was due to lower concentrations of apolipoproteins of LDL and VLDL particles. In contrast to this, choline, phosphocholine and GPC as well as cholesterol, 5α-cholesterol and 7-ketocholesterol were higher in patients. Components of the tricarboxylic acid cycle, namely citrate and α-ketoglutarate, were also increased in AS men. The same applied to creatinine, uridine and the amino acids Cys, Gln, Glu and His and the biogenic amine taurine. An increase in taurine in semen with less motile sperm is astonishing, because taurine has often been described to be positively correlated to sperm motility. The authors explained this higher amount with the possibility of a diet rich in sulfur-containing amino acids. Phe and Tyr as well as cytidine were decreased in patients. Due to the fact that Phe is further metabolized to Tyr, a precursor of dopamine which has been shown to improve sperm motility in several species [25,26], this result seems reasonable.

The SP of 31 OAT and 28 proven-fertile men attending a Turkish in vitro fertilization (IVF) unit was investigated by ^1^H NMR on a 600 MHz Bruker Avance III HD NMR spectrometer [27]. Hormone levels (FSH, LH, prolactin, testosterone) did not differ between the study groups, but sperm concentration, motility and morphology were below the WHO reference values in OAT patients and differed significantly from controls. The separation of OAT samples from controls using principal component analysis (PCA) and partial least-squares discriminant analysis with variable importance in the projection (PLS-DA-VIP) plot was based on lactate, citrate, creatinine, α-ketoglutarate, putrescine, spermine, Arg, Gln, Lys, Tyr and Val. The levels of these metabolites were decreased in OAT patients, except for Tyr, which was increased in patients compared to controls. A *t*-test analysis revealed significantly different concentrations of choline, citrate, α-ketoglutarate, putrescine, spermine, Tyr and Val in a comparison of both cohorts.

^1^H NMR spectra of SP from men with and without varicocele were acquired on a Varian VNMRS400 spectrometer operating at 400 MHz [28]. Broad resonances were suppressed by making use of the spin-echo sequence with τ = 70 ms. Semen samples of 24 proven fathers without varicocele (C), 21 proven fathers with varicocele (VF) and 35 subfertile men with varicocele (VI) were centrifuged to separate sperm cells from SP. The VI group was characterized by poor spermiograms (low sperm concentration, low sperm count, low motility) and high blood FSH and LH levels compared to the C and VF groups. The percentage of bilateral varicocele was 60% in VI vs. 14% in VF men. Concentrations of analytes detected by ^1^H NMR are given as discrete concentrations but only as high, medium and low. The results might seem somewhat confusing because they are not in accordance with the semen parameters. The relative concentrations of some analytes were high in C and low in VF but were in-between C and VF in VI. The most important variables for group discrimination that have been assessed as high in C, low in VF and medium in VI were caprate (the salt of decanoic acid) and uridine as well as the amino acids Gln, Ile and Tyr. It is not clear why just C10:0 and no other fatty acids with longer or shorter chains were identified. Those that were low in C, high in VF and medium in VI were 4-aminobutyrate, citrate, glycosides, lactate, 3-hydroxybutyrat and the amino acids N-acetyl-Tyr and Val. Due to these results, one can speculate that the disturbed blood flow itself has more impact on the metabolome of the SP than the low semen quality. The only metabolite that followed the logical order of being “medium” in VF was Arg with the lowest relative concentration in VI and highest concentration in C. Thus, the Arg concentration could indeed account for low semen quality.

In 2020, Murgia et al. investigated SP samples of 47 subjects attending a fertility center using a 500 MHz NMR spectrometer from Agilent Technologies [29]. The metabolome status predominantly excluded lipids because only the upper aqueous layer of a Bligh–Dyer extraction was further processed. This workup is a bit unusual because this type of extraction is normally used to enrich the apolar compounds (particularly lipids) in a sample. The authors divided the cohort into normal controls (*n* = 29) and oligozoospermic samples (*n* = 18) and referred to the guidelines of WHO 2010 [3]. Astonishingly, the table provided within the manuscript does not indicate oligozoospermia, which would mean only ejaculates with a total of less than 39 × 10^6^ cells were found. However, the mean was 48.6 × 10^6^ cells in the supposed oligozoospermic group. A standard deviation is missing. Furthermore, all samples, the controls and the supposed oligozoospermic samples, seem to be AS, as the progressive motility was far below 32% in both groups (18.6 ± 9% and 6.7 ± 10%, respectively). Total motility in the supposed oliozoospermic group was 26.8 ± 23%, which (together with the aforementioned aspects) rather points to AS than oligozoospermia. Despite these flaws, the authors identified 34 metabolites by ^1^H NMR with Asp, choline, fructose and myo-inositol showing the most pronounced differences between the two study groups. The concentrations of Asp and myo-inositol were higher in the controls, while those of choline and fructose were lower. Additionally, the fructose and Asp levels correlated with the total number of sperm and the total motile sperm cells in a negative and a positive manner, respectively. The myo-inositol level correlated positively with the percentage of progressively motile sperm. Because of the unclear classification of the samples, this study is not included in Table 2.

A study investigating the SP of 14 teratozoospermic patients vs. 15 proven fertile controls with ^1^H NMR on a Bruker DRX 500 MHz NMR spectrometer [30] identified 18 metabolites with different numbers in patients and controls. Citric acid, choline, glucose, lactate, myo-inositol, Ala, Gln, Ile, Leu, Pro, Leu, Lys, Thr, Tyr and Val were higher, whereas Glu, cholesterol and taurine were found at lower concentrations in teratozoospermic patients. According to the authors’ statements, low concentrations of taurine, an important antioxidant, might result in lower antioxidative activity of SP and, thus, to male factor infertility. One problem of this study might be the freezing procedure used for the ejaculates, which were frozen to −80 °C after collection without any separation of cells and SP. Thus, sperm cells were most likely damaged due to disruption of the membrane, and results might not only represent the status of the SP but might be influenced by metabolites released from damaged sperm cells.

Alipour and colleagues [9] used ^1^H-NMR to compare human SP from 31 males with normal spermiogram parameters from couples undergoing either IVF or ICSI after long- and short-term abstinence. TSP was used as the internal standard. Alipour and coworkers found 30 metabolites, among which 9, namely fructose, pyruvate, acetate, choline, methanol, N-acetylglucosamine, O-acetylcarnitine, uridine and GPC, were differentially concentrated between samples of men with long- and short-term sexual abstinence. They related the number of the metabolites in SP to 1 × 10^6^ sperm and found that the moieties of pyruvate, an important source of energy, and taurine, an amino acid that has been described in the context of sperm motility, were higher after short-term abstinence. The authors concluded that these elevated total amounts are probably the reason for higher sperm motility rates in second ejaculates, which has been shown in this as well as in other studies. A positive impact on pregnancy outcome has also been associated with a shorter period of sexual abstinence due to improved spermiogram parameters (recently reviewed in [31]). A cautionary note is necessary here: the presence of methanol is very strange and not at all explained by the authors. This raises the question of whether (a) all resonances were correctly assigned in this study or (b) methanol actually represents an impurity.

**Table 2:** Metabolites for pathological conditions in the context of male fertility identified by proton nuclear magnetic resonance. For the discriminatory metabolites, the direction of observed changes is illustrated by arrows. OAT—oligoasthenoteratozoospermia, SP—seminal plasma.

Ref.	Pathological Condition/ Study Group 1	Controls/ Study Group 2	Sample	Discriminatory Metabolites
[19]	azoospermia (spermatogenic failure, *n* = 21)	normozoospermic	SP	↓ lactate ↓ GPC ↓ citrate
	obstructive azoospermia (vasectomy, *n* = 14)			↓ lactate ↓ GPC
	severe OAT (*n* = 7)			
[20]	azoospermia (spermatogenic failure, *n* = 58)	obstructive azoospermia (vasectomy, *n* = 17)	SP	↑ ratio choline/citrate ↑ ratio choline/lactate ↑ ratio GPC/choline
	azoospermia, normal FSH (spermatogenic failure, *n* = 9)	obstructive, azoospermia, normal FSH (vasectomy, *n* = 7)	SP	↑ ratio choline/lactate ↑ ratio GPC/choline
[21]	idiopathic infertility (normozoospermic, *n* = 65)	normozoospermic proven fathers (*n* = 60)	SP	↓ Ala ↓ citrate ↓ GPC ↑ Phe
	oligozoospermia (*n* = 60)			↓ citrate ↓ GPC ↑ Phe
[23]	idiopathic infertility (normozoospermic, *n* = 17)	normozoospermic proven fathers (*n* = 6)	SP	↑ fructose ↑ hippurate ↑ 2-hydroxyisovalerate ↑ amino acids Lys, Val
	oligozoospermia (*n* = 20)			↓ guanidoacetate ↓ fructose
	AS (*n* = 20)			-
	teratozoospermia (*n* = 20)			-
	azoospermia (*n* = 20)			↓ guanidoacetate ↓ fructose
[27]	OAT (*n* = 31)	normozoospermic (*n* = 28)	SP	↓ amino acids Arg/Lys, Gln, Val, ↓ citrate ↓ choline ↓ creatinine ↓ α-ketoglutarate ↓ lactate ↓ spermine/putrescine ↑ amino acid Tyr
[30]	teratozoospermia (*n* = 14)	normozoospermic proven fathers (*n* = 15)	SP	↓ cholesterol ↓ amino acid Glu ↓ taurine ↑ amino acids Ala, Gln, Ile, Leu, Lys, Pro, Thr, Tyr, Val ↑ choline ↑ citrate ↑ D-glucose ↑ lactate ↑ myo-inositol ↑ pyruvate
[9]	short-term abstinence (2 h), IVF/ICSI couples, ≥15 × 10^6^ sperm/mL (*n* = 31)	long-term abstinence (4–7 d), IVF/ICSI couples, ≥15 × 10^6^ sperm/mL (*n* = 31)	SP	↓ fructose ↓ acetate ↓ choline ↓ methanol ↓ N-acetylglucosamine ↓ O-acetylglucosamine ↓ uridine ↓ GPC ↑ pyruvate

### 2.2. Studies Based on Raman Spectroscopy

Both IR and Raman spectroscopy are suitable methods for monitoring vibrations on a molecular level. The energy to bend, stretch or distort a bond depends on the force constant of the linkage and can be used to monitor the presence or absence of dedicated functional groups. Raman spectroscopy is more suitable for monitoring less polar functional groups such as C-C or C-H, while IR is the method of choice to detect polar residues such as C=O or O-H. Although there are methods (such as attenuated total reflection (ATR)) which enable the suppression of water [32], the intensity of the residual water is a significant problem in IR—particularly if the spectra have to be quantitatively analyzed. Because Raman spectroscopy is less sensitive to water, it is more readily applicable to biological samples.

Gilany and coworkers used ATR-IR spectroscopy and Fourier transformed IR (FT-IR) spectroscopy to detect abnormalities in the metabolome of SP due to altered spermatogenesis [33]. This study only included ten samples (normozoospermic and azoospermic); six were used for ATR-IR and four for FT-IR. They found that ATR-IR without any further extraction is not suitable for the investigation of SP due to its high water content. Obviously, the O-H vibration suppresses other signals and/or irregularities in the baseline, making determinations of metabolites challenging. Therefore, the authors precipitated proteins from SP by methanol–water (9:1, *v*/*v*) and re-suspended the dried supernatant in chloroform before recording FT-IR spectra. Significant patterns for normozoospermic and azoospermic men were found in the fingerprint region (C-Cl, C-O, C-N, C-C vibrations). Of course, the assessment of azoospermia by spermiogram analysis is quite simple and does not require additional analytical methods. Nevertheless, the molecular changes in any semen abnormality are interesting to know in order to obtain hints about the cause of the pathology.

The same authors combined Raman spectroscopy with advanced methods of data analysis to distinguish between the SP metabolome of asthenospermic (low sperm motility) and normozoospermic men after protein precipitation [34]. The authors present a classification model, which reportedly arose from ten samples per group. Unfortunately, the training set of normospermic men is not visible in the graph and the reliability of the model was only tested for three samples of each group with one false prediction. Further, there are no metabolites described in this article. In another paper, Gilany and other coworkers used Raman spectroscopy to distinguish between 15 fertile, 10 azoospermic testicular sperm extraction (TESE)-positive and 10 azoospermic TESE-negative patients [35]. All samples were investigated in triplicate. Applying PCA, the patients were split into separate groups with an overlap between fertile controls and TESE-positive men. TESE-negative patients were further subdivided into three groups that corresponded to hypospermatogenesis, maturation arrest and germinal aplasia. Furthermore, an oxidative imbalance was identified for TESE-negative samples using the –CH functional group of Raman spectra, although no detailed information was provided for how this approach was achieved. Nor did the reference to the prior publications [7,36] explain why the C-H vibration is useful as an oxidative stress biomarker.

### 2.3. Studies Based on Liquid Chromatography–Mass Spectrometry

Liquid chromatography—mass spectrometry (LC/MS) is presumably the most powerful approach to characterize complex biological samples. It is also increasingly used as an investigative tool in the clinical laboratory. Although a more detailed description of this method is out of the scope of this review, one comment seems necessary. In the majority of investigations, “Q” (quadrupole) mass spectrometers are used. Quadrupole devices are very common. For targeted metabolomics, triple-quad devices are used. The first quadrupole is used to select the ion of interest. In the second quadrupole, the ion is fragmented in order to obtain useful fragment ions (MS/MS), and the third quadrupole is used to separate the fragment ions from each other. For nontargeting screening, the quadrupole might be used for a preselection of analytes, but the identification is achieved by time-of-flight (TOF), ion cyclotron resonance (ICR) or orbitrap (selection by ion trap). The less experienced reader will probably ask why the previous separation of the mixture is necessary prior to detailed MS analysis. This is necessary because different analytes have different tendencies to generate ions, and multiple ions are detected by MS at the same time. In complex mixtures, this may lead to the suppression of selected analyte classes. This aspect was comprehensively discussed recently [37].

Chen and colleagues [38] compared SP samples of 18 patients suffering from Kidney-Yang Deficiency Syndrome (KYDS), a syndrome described in traditional Chinese medicine, with primary and secondary manifestations. Primary manifestations of patients included in this study were an abnormal spermiogram and erectile or ejaculation disorders. By applying LC-QTOF-MS to protein-precipitated supernatants of SP, the authors found 41 differentially concentrated metabolites. One problem of this study is that 14 out of 18 patient ejaculates were either oligozoospermic, AS or teratozoospermic and from men suffering from KYDS. The control group, however, consisted of completely healthy men. Thus, the impact of KYDS on the SP metabolome remains elusive. Furthermore, the results raise some concerns, e.g., the detectability of molecules in the respective ion mode. In the majority of cases, acidic molecules are much better detectable in the negative-ion mode, and, thus, the detection of deoxycholate, phenylpyruvate and taurocholate, etc., in the positive-ion mode is uncommon. Regarding these issues, there will be no deeper interpretation of this study.

In 2019, we published a study using the Absolute IDQ p180 kit (Biocrates Life Sciences AG, Innsbruck, Austria) for targeted metabolomics of sperm and the SP of healthy men by LC-MS to unravel the normal concentrations of approximately 180 metabolites in these specimens [11]. These metabolites were amino acids, biogenic amines, acylcarnitines, lysophosphatidylcholines (LPC), phosphatidylcholines (PC), sphingomyelins and the sum of hexoses. Additionally, the metabolite concentrations were correlated with the spermiogram parameters. In sperm and in SP, 171 and 177 out of 180 metabolites could be detected, respectively. Correlation analyses revealed that the concentrations of most sperm metabolites correlated with sperm motility. Here, the highest positive correlation was found for taurine, which may emphasize the beneficial effects of taurine as an antioxidant. On the contrary, the concentrations of most of the detected metabolites from SP correlated with sperm concentration and morphology. The above-mentioned Biocrates Kit is unequivocally an excellent method for the determination of many defined metabolites, which include various sperm and SP-related metabolites, because it contains many standards in a known concentration. However, this kit is rather expensive, and, thus, the investigation of big cohorts is very costly.

In the same year, Yu and colleagues applied a targeted approach to the SP of healthy and AS individuals with a focus on arachidonic acid (C20:4) and metabolites derived thereof [39]. The study groups showed a progressive sperm motility of >50% and <50% in computer-assisted semen analysis (CASA), respectively. Unfortunately, there is no information on the morphology of sperm, which should be mentioned due to different pathologies involving sperm motility and morphology. Samples were analyzed by HPLC-ESI-MS/MS on a Shimadzu HPLC-20A and an AB Sciex API 4000 quadrupole ion trap (QTRAP) MS/MS system, both working in the negative-ion mode. Concentrations of C20:4, 15-HETE, 8,9-EET, 14,15-DHET, 14,15-EET, 5-HETE, tetranor-PGE metabolite (the urinary metabolite of PGE1 and PGE2), 11,12-DHET and 20-HETE were significantly higher in the SP of AS compared to normozoospermic men. Concentrations of the oxidative stress metabolites PGE2, PGD2 and PGF2α were significantly lower in abnormal samples. Additionally, the authors showed that C20:4 is capable of inducing the P38-MAPK pathway in sperm and concluded that an abnormal C20:4 metabolic network can reduce sperm motility via P38-MAPK activation. A review about the relevance of the mentioned arachidonic-acid-derived compounds is available in [40].

Metabolites of methanol-precipitated SP and their correlation to semen quality as well as environmental pollution were studied by Huang and coworkers [41]. They grouped 98 participants into quartiles according to their semen parameters. According to the figures showing the scoring plots of a PLS-DA model, there were 24 men with high semen quality (first quartile) and 23 with low semen quality (fourth quartile). Analysis of the samples using a Waters™ ACQUITY UPLC system equipped with an HSS T3 column and coupled to a Q Exactive orbitrap mass spectrometer from Thermo Fisher revealed 5232 metabolic features in the positive- and 3496 in the negative-ion mode. For our review, the results on the pollutants are neglected. After adjustment for age, body mass index (BMI), abstinence time, smoking and drinking habits, the concentrations of only a few molecules were associated with either sperm count (*n* = 22) or sperm concentration (*n* = 16). Unfortunately, the adjustment/normalization of the data remains unclear. It is not clear whether only BMI-age-matched samples were taken into account or if there was any other procedure. Furthermore, it is not clear to us why the fold changes of these molecules were separated into “sperm count” and “sperm concentration” (and were slightly different in these groups). The authors reported that the concentrations of 14 metabolites were higher in the low-quality group compared to the high-quality group and the concentrations of other 14 metabolites were lower. For details, please see Table 3.

A study using UPLC-MS/MS was published in the same year by Chen et al. [42]. They investigated small-molecule metabolites in the SP (protein-depleted by precipitation with organic solvents) of 77 men whose progressive sperm motility was <32% (AS) and compared the results with samples from 63 normozoospermic men on an ACQUITY I-Class UPLC equipped with a XEVO TQS-micro triple-quadrupole MS from Waters^TM^. For the separation of polar compounds, an HSS (reversed-phase) C18 column (Waters^TM^, Milford, MA, USA) was used at 40 °C. Additionally, they performed quantitative real-time polymerase chain reaction (qPCR) to unravel the expression of certain enzymes in 10 sperm samples per group on an ABI StepOnePlus Real-time PCR System (Applied Biosystems, Waltham, MA, USA). Twenty-seven metabolites were identified in SP with the respective standard substances, nineteen of them being indicative of AS. The concentrations of metabolites associated with the tricarboxylic acid (“citrate”) cycle, namely pyruvate, succinate, malate and citrate, were significantly lower in AS patients compared to controls. The level of lactate (as one potential product of the glucose metabolism) was dramatically increased in patients. This indicates a considerable energy consumption by the cells under anaerobic respiration. In addition, spermine, ornithine and metabolites involved in tyrosine metabolism (Phe, Tyr), purine metabolism (hypoxanthine, inosine), branched-chain amino acid metabolism (Val) and the Methionine cycle (Met) were lower in the SP of AS patients compared to that of controls. In accordance with their findings from UPLC, Chen and colleagues found decreased gene expressions of spermine synthase (27% of control), fructokinase-2 (37%), citrate synthase (47%) and succinate dehydrogenase (54%) in the sperm of AS patients.

Only a few months later, another Chinese group published their results on a similar study using the same equipment, but a column temperature of 25 °C, also investigating the SP of AS men (*n* = 20) compared to controls (*n* = 20) [43]. Healthy controls were assigned based on normal CASA results. Since PCA did not result in a clustering of both groups, PLS-DA was performed and resulted in a feasible differentiation of both groups. Nine differentially concentrated metabolites were identified that correlated with sperm motility. Among these, the concentrations of creatinine, uric acid, N6-methyladenosine (m6A), uridine and taurine were higher, whereas free carnitine (C0), carnitine C16:0, nicotinamide and N-acetylputrescine were lower in AS patients compared to controls. The reason for the elevated taurine level in AS patients remains elusive, and the explanation of a taurine-rich diet of the AS patients is speculative. Despite this, lower carnitine levels are in accordance with carnitine supplementation in AS treatment. The authors further suggested the m6A SP level as a biomarker for AS, because it had already been shown to be increased in the sperm of AS men.

In the same year, Wang and colleagues investigated the SP metabolome of 660 volunteers in relation to semen quality and urinary phthalate metabolites using LC coupled to a Q Exactive high-resolution mass spectrometer from Thermo Scientific [44]. Samples were grouped into poor (*n* = 140), intermediate (*n* = 300) and high semen quality (*n* = 220) according to their performance in semen analysis. In the positive-ion mode, 2770 metabolic features were detected vs. 2640 in the negative-ion mode. Despite the correlation with phthalate (used as plasticizers), the authors described potential biomarkers of poor semen quality. After adjustments for age, BMI, creatinine, sexual abstinence time, smoking status and being a father, concentrations of 24 metabolites were shown to be increased in the SP from poor-semen-quality samples and only 1, namely oleamide, was found in lower concentrations. Those increased were GPC, adenine, epitestosterone, 11b-hydroxyprogesterone, (9S,10S)-9,10-dihydroxyoctadecanoate, 7β,12α-dihydroxykaurenolide, 6-keto-PGF1 α, 8-iso-15-keto-PGF2α, PGE2, L-3-phenyllactate, hydroxyphenyllactate, xanthine, 12,13-dihydroxy-9-octadecaenate, the amino acids Arg and His, the fatty acids C18:1, C22:5 and C22:6 and the carnitines C0, C2, C3, C6, C16 and C18:2.

Another paper investigated SP from 35 proven fathers and 140 infertile patients using an LC-30AD UHPLC system from Shimadzu with ACQUITY UPLC BEH HILIC and HSS C18 columns from Waters™ [45]. The patients were grouped into OAT (*n* = 20), AT (*n* = 32), AS (*n* = 76), azoospermic (*n* = 7) and OA (*n* = 5) samples. Even though the authors stated that samples were classified according to the WHO criteria, it is not clear why the fourth edition [2] and not the then-valid fifth edition [3] was used for a study performed in 2019. Furthermore, it is not clear what “strong vital sperm” are, and the authors defined <50% normomorphic sperm as teratozoospermia. However, the fourth edition of the WHO manualstates that “data from assisted reproductive technology programs suggest that, as sperm morphology falls below 15% normal forms [2] the fertilization rate in vitro decreases.” Looking at the mean values and the standard deviations of the groups, e.g., sperm number (×10^6^) of OAT (47.58 ± 30.77) and OA (47.3 ± 68.67) men, it seems that the limit for sperm count for oligozoospermia (<40 × 10^6^) was not used consistently. The authors used an unusual sample preparation protocol. After separation from sperm cells, aqueous and organic molecules of the SP were separated using methanol–chloroform–H_2_O (2:1:1, *v*/*v*/*v*). Then, the two phases underwent solid-phase extraction to collect the hydrophilic amines and the hydrophobic amines/alcohols. Metabolites were then derivatized (for details, see the supplemental material of [45]) before being analyzed by LC-MS. Using this method, 642 metabolic features were detected. Identified molecules were correlated to spermiogram parameters, resulting in positive correlations of sperm concentration with carnitine and negative correlations of this parameter with creatine riboside and butoconazole. Progressive motility was positively associated with lyso-SM(d18:0). Sperm deformity correlated positively with glutamylarginine, leucylproline, Val, prostaglandin E3, C18:1, C20:4, different carnitines and hypoxanthine. The authors explained the occurrence of oligopeptides by the degradation of peptides or protein hormones. Negative correlations indicative of sperm deformity were found to correlate with creatine riboside, butoconazole, γ-glutamyl-methylselenocysteine, isopentylpyrophosphate, 2-phosphoglycerate and propylphenylalanine and, for sperm motility, with spermine. Thus, the statement that the “result agreed well with previous study of positive correlation between concentrations of spermine and motility of ejaculated spermatozoa” seems strange.

One study investigated whether there is a relationship between semen factors and unexplained recurrent spontaneous abortion (URSA) [46]. The study included seminal fluid from 28 male partners in the URSA group and 25 matched controls (fathers of at least one healthy child). Spermiogram parameters were surveyed according to the WHO criteria (fifth edition [3]). Proteins from SP and sperm (lysed by ultrasound for 1 min) were precipitated with methanol. LC was performed on a UPLC Ultimate 3000 system (Dionex, Sunnyvale, CA, USA) with a Q-Exactive mass spectrometer (Thermo Fisher Scientific, Waltham, MA, USA) and resulted in 120 and 136 metabolites in sperm and SP, respectively. In sperm, the levels of 3-phenylbutyrate, cholesterol and hexadecanedioate were higher in the URSA group compared to healthy controls, while pyroglutamate was found in much lower concentrations. In SP, 2-hydroxycaproate, ascorbate, neopterin, glyocolate, *N*-oleoylethanolamine, taurodeoxycholate and xanthosine were increased, whereas guanine was decreased in the URSA group. Sperm concentration, total sperm count, motility and normal morphology were significantly decreased in men from URSA couples. The authors concluded that a reduced concentration of pyroglutamate, an intermediate of the gluthathione metabolism, might increase the risk of URSA due to disturbed antioxidative mechanisms and increased reactive oxygen species generation. Additionally, a disturbed guanine metabolism might contribute to URSA, as related mouse experiments showed fewer offspring in affected animals.

Only recently, Boguenet et al. [47] published a study also using the metabolomics Absolute IDQ p180 kit from Biocrates on SP samples from 20 men with severe OA and 20 men with normal semen parameters. All OA patients underwent ICSI, whereas the controls underwent intrauterine insemination (*n* = 8), IVF (*n* = 11) or ICSI (*n* = 1). Unfortunately, there is no further explanation for why the control males underwent assisted reproduction. The authors detected 110 out of 180 metabolites and found 37 discriminant metabolites between the study groups. Among them were carnitine, five acylcarnitines, six amino acids, four biogenic amines, four sphingomyelins, 13 acyl-acyl PC and four alkyl-acyl PCs. They found Glu, the amino acid that is most abundant in healthy subjects and that shows a positive correlation with sperm count [11], being reduced in the SP of OA patients. A lower spermine/spermidine ratio, a measure for the activity of spermine synthase, matched with a lower sperm motility, a correlation that has been described before [11,45]. Interestingly, the levels of PC containing polyunsaturated fatty acids (PUFAs) were significantly lower in OA patients compared to the control group. It is well-known that PUFAs are important for sperm motility and sperm membrane fluidity [48] and are incorporated into the cell membrane during the transit of the spermatozoa through the epididymis.

Lipidomics, the analysis of the lipidome of a certain biological sample, is considered as an important subfield of metabolomics [49,50]. Additionally, as already mentioned above, the metabolome of sperm could give even more hints about the reasons for male infertility than the metabolome of SP. Lately, it has been reported that the sperm lipid composition varies between sperm from normozoospermic and AS samples [51]. In this study, samples from 12 AS and 12 normozoospermic men were investigated by targeted lipidomics using the ExionLC coupled to a QTRAP 6500 Plus, both from Sciex. Normal-phase HPLC was used for polar lipids (phospholipids, sphingolipids) and reverse-phase HPLC for neutral lipids (triacylglycerols, diacylglycerols, cholesteryl esters). Total cholesterol and cholesteryl esters were analyzed by HPLC/APCI/MS/MS because these apolar compounds only result in a relatively poor ESI response [52]. Prior to MS, sperm were separated from SP by density gradient centrifugation and sperm lipids were extracted with chloroform–methanol. In total, among 479 identified lipid species, the concentrations of 48 lipids were significantly altered in sperm from AS samples compared to those from normozoospermic samples. Among them were 9 PE (higher in AS), 16 cardiolipins (CL, higher), 6 monosialodihexosyl gangliosides (GM3, higher) and 7 triacylglycerol (TAG) molecules (lower). Concentrations that correlated with progressive motility were the sum of GM3 and lysophosphatidylinositols (LPI), the GM3 species d18:1/16:0, d18:1/22:0, d18:1/24:0, d18:1/24:1, d18:0/22:0 and d18:0/24:0, LPI 18:0, PE 32:1, PE 34:1, PE P-38:6 and TAG 52:1. Total motility was additionally associated with the total amounts of cholesterol, PE and alkenyl-acyl PC, PC P-40:4, PE P-38:4, PE P-38:6, PE P-40:6, PE 38:6, PE 40:6 and PG 38:4. The latter represent sum formulas of the number of C atoms and double bonds. For an update on lipid nomenclature, see [53].

The biochemical assessment of amino acids in SP among 50 azoospermic, 50 OAT and 50 healthy control samples [54] was performed using an S433 amino acid analyzer and an HPLC-based system from Sykam GmbH (Eresing, Germany). This revealed significantly lower median concentrations of Ala, Arg, Asn, Asp, Gly, His, Ile, Leu, Lys, Orn, Pro, Ser, Thr, Trp, Tyr and Val in azoospermic men compared to the other two groups. Taurine and α-amino adipic acid (α-AAA) were lower in both patient groups compared to controls. OAT samples were also lower in Ala, Asn, Asp, His, Leu, Lys and Pro in comparison to the control samples. Additionally, the study reported lower Zn^2+^ levels determined via a colorimetric assay in azoospermic SP. Blood FSH and LH were much higher in azoospermic patients than in the other cohorts, whereas testosterone and prolactin did not differ significantly.

Just recently, we published a small study on the influence of smoking on caspase activity and on the levels of defined metabolites in mature sperm cells and SP using LC-MS [55]. As a study population, we recruited ten smoking and ten nonsmoking normozoospermic men. Semen samples were separated by density gradient centrifugation. Sperm cells were lysed by methanol–water (1:1) and ultrasonication on ice. Targeted metabolomic investigations were again performed using the AbsoluteIDQ p180 kit from Biocrates. Analyses were acquired on a QTRAP mass spectrometer (ABI Sciex API 5500 QTRAP). Amino acids and biogenic amines were analyzed by LC-MS/MS. The MS was coupled to a UPLC system from Waters™. Acylcarnitines, lipids and the sum of hexoses were investigated with flow injection analysis–MS/MS. Smokers and nonsmokers were nicely separated by PCA. In the PCA plot, smokers with a lower cigarette consumption appeared closer to the nonsmoking population than those men who consumed more cigarettes. Even though there were no differences in the spermiograms of the two groups, caspase-3 activity in sperm cells showed a high positive correlation with cigarette consumption, and the concentrations of some amino acids, biogenic amines and (acyl)carnitines were altered in sperm cells of smoking individuals. Furthermore, some metabolites were only detected in either smokers or nonsmokers, and some metabolites could not be detected in any of the samples. This might have been due to either the absence or a very low concentration of the molecule. Limitations of the study were definitely the small number of participants (due to the high costs of the kit) and that men were not proven fathers.

**Table 3:** Metabolites for pathological conditions in the context of male fertility identified by LC-MS. For the discriminatory metabolites, the direction of change is indicated by arrows. AS—asthenozoospermia, AT—asthenoteratozoospermia, OA—oligoasthenozoospermia, OAT—oligoasthenoteratozoospermia, SP—seminal plasma.

Ref.	Pathological Condition/ Study Group 1	Controls/ Study Group 2	Sample	Discriminatory Metabolites
[39]	AS (*n* = 30)	normozoospermic (*n* = 33)	SP	↓ PGE2, PGD2, PGF2α ↑ 11,12-DHET ↑ 8,9-EET, 14,15-EET ↑ fatty acid C20:4 ↑ 5-HETE, 15-HETE, 20-HETE ↑ tetranor-PGEM
[41]	low-quality semen (*n* = 23)	high-quality semen (*n* = 24)	SP	↓ α-AAA ↓ 5-aminoimidazol-riconucleotide ↓ amino acid Lys ↓ capryloylglycine ↓ carnitines C0, iso-C4, C6-OH, pivaloylcarnitine ↓ fatty acid C22:6 ↓ glutaconate ↓ GPC ↓ imidazole-4-acetaldehyde ↓ lyso-SM(d18:1) ↓ tocotrienol ↑ N-acryloylglycine ↑ carnitine C3-DC ↑ fatty acid C22:4 ↑ G6-P ↑ 11b-hydroxyprogesterone ↑ imidazoleacetate riboside ↑ 3-oxohexanoate ↑ PGB2, PGE2 ↑ PS ↑ dipeptide Trp-Asp ↑ tyramine glucoronide ↑ ubiquinone-2 ↑ uracil
[42]	AS (*n* = 77)	normozoospermic (*n* = 63)	SP	↓ amino acids Arg, Met, Phe, Pro, Trp, Tyr, Val ↓ aminobutyrate ↓ citrate, malate, pyruvate, succinate ↓ hypoxanthine, inosine ↓ nucleobases adenine, cytosine ↓ spermine ↑ lactate ↑ Orn
[44]	low-quality semen (*n* = 140)	high-quality semen (*n* = 220)	SP	↓ oleamide ↑ adenine, xanthine ↑ amino acids Arg, His ↑ carnitines C0, C2, C3, C6, C16, C18:2 ↑ (*S*,*S*)-9,10-dihydroxyoctadecanoate ↑ 7β,12α-dihydroxykaurenolide ↑ 12,13-dihydroxy-9-octadecenoate ↑ epitestosterone, 11b-hydroxyprogesterone ↑ fatty acids C18:1, C22:5, C22:6 ↑ GPC ↑ 6-keto-PGF1 α, 8-iso-15-keto-PGF2α, PGE2 ↑ L-3-phenyllactate, hydroxyphenyllactate
[45]	AS (*n* = 76)	proven fathers (*n* = 35)	SP	↓ dipeptides Leu-Pro, Pro-Gly, Glu-Arg ↓ amino acid Val ↑ 2-phosphoglycerate ↑ creatine riboside ↑ isopentenylpyrophosphate ↑ *γ*-glutamyl-methylselenocysteine
	AT (*n* = 32)			↓ butoconazole ↓ carnitines C0, C3, C6, C22:5, pivaloylcarnitine ↓ dipeptide Gly-Phe ↓ LPC 20:0, LPE 16:0, PE P-16:0 ↓ lithocholate ↓ PGE3 ↑ dethiobiotin ↑ dipeptide Tyr-Glu
	OAT (*n* = 20)			↓ dethiobiotin ↓ dipeptides Pro-Gly, Lys-Gly, Val-Ser, Pro-Phe ↓ fatty acids C18:1, C20:4, C20:0(2OH) ↓ 6-methylnicotinamide ↓ methylpyrrolo [1,2-a]pyrazine ↓ nonanol ↓ piperanine ↓ *N*-oleoylethanolamine ↑ capsiamide ↑ hypoxanthine ↑ amino acid His ↑ lyso-SM(d18:0) ↑ palmitic amide ↑ penmacric acid ↑ spermine
[46]	men from URSA couples (*n* = 28)	proven fathers (*n* = 25)	sperm	↓ pyroglutamate ↑ cholesterol ↑ 3-phenylbutyrate ↑ hexadecanedioate
			SP	↓ guanine ↑ 2-hydroxycaproic acid (2-hydroxyhexanoate) ↑ ascorbate ↑ neopterin ↑ glycocholate ↑ *N*-oleoylethanolamine ↑ taurodeoxycholate ↑ xanthosine
[47]	OA (*n* = 20)	normozoospermic (*n* = 20)	SP	↓ amino acids Ala, Asp, Glu, Met, Pro, Trp ↓ biogenic amines alpha-AAA, serotonin, spermine, spermidine ↓ carnitines C0, C3, C5, C5:1, C5-OH, C6 (C4:1-DC) ↓ sphingolipids SM C16:1, SM (OH) C14:1, SM (OH) C16:1 ↓ acyl-acyl phospholipids PC 28:1, PC 34:2, PC 36:2, PC 36:3, PC 36:4, PC 38:0, PC 38:3, PC 38:4, PC 38:5, PC 38:6, PC 40:4, PC 40:5, PC 42:5 ↓ alkyl-acyl phospholipids PC O-34:0, PC O-40:5, PC O-40:6
[51]	AS (*n* = 12)	normozoospermic (*n* = 12)	sperm	↓ LPS 18:1 ↓ DAG 32:0, DAG 34:1, DAG 36:1 ↓ TAG 48:1, TAG 48:0, TAG 50:1, TAG 50:0, TAG 52:2, TAG 52:1 ↑ Cer d18:1/15:0 ↑ CL 66:4, CL 68:6, CL 72:9, CL 74:10, CL 74:9, CL 74:8, CL 74:7, CL 76:10, CL 76:9, CL 78:12, CL 78:11 ↑ total cholesterol, total GM3, LPI, PE ↑ GM3 d18:1/16:0, GM3 d18:1/22:0, GM3 d18:0/22:0, GM3 d18:1/24:1, GM3 d18:1/24:0, GM3 d18:0/24:0 ↑ LPI 18:0 ↑ acyl-acyl phospholipids PE 32:1, PE 34:1, PE 38:6, PE 38:3, PE 40:6, PE 40:5, PG 38:4 ↑ alkenyl-acyl phospholipids PC P-38:3, PC P-40:6, PC P-40:4, PE P-38:6, PE P-38:4, PE P-40:6
[54]	nonobstructive azoospermia (*n* = 50)	proven fathers (*n* = 50)	SP	↓ amino acids Ala, Arg, Asn, Asp, Gly, His, Ile, Leu, Lys, Orn, Pro, Ser, Thr, Trp, Tyr, Val ↓ biogenic amines α-AAA, taurine ↓ Zn^2+^
	OAT (*n* = 50)			↓ amino acids Ala, Asn, Asp, His, Leu, Lys, Pro ↓ biogenic amines α-AAA, taurine
[55]	smoking, normozoospermic (*n* = 10)	nonsmoking, normozoospermic (*n* = 10)	sperm	↓ biogenic amines ADMA, serotonin ↓ carnitines C7-DC, C8, C10, C10:2, C12-DC, C14, C14:1, C14:1-OH, C14:2-OH, C16:1, C16:2, C16:2-OH ↓ ratios ADMA/Arg, total DMA/Arg, Tyr/Phe, spermidine/putrescine, (C16 + C18)/C0 ↑ amino acids Asp, Gln, Gly, Phe, Val ↑ ratios Cit/Arg, spermine/spermidine, C2/C0, (C2 + C3)/C0

### 2.4. Studies Based on Gas Chromatography

Gas chromatography (GC) offers the very best resolution among all chromatographic techniques and, thus, is widely used [56]. GC relies on the volatility of the sample, which means that analytes of choice have to be transferred into the gas phase without decomposition. This applies only for a small number of analytes, and derivatization to enhance the volatility is regularly required. This makes GC normally more time-consuming than HPLC.

In 2001, Gulaya et al. reported that the levels of the polyunsaturated fatty acids eicosapentaenoic and docosahexaenoic acid, which usually provide fluidity to the sperm membrane, were significantly lower in men with a higher number of abnormal sperm (*n* = 16) compared to proven fathers (*n* = 8) [48]. Additionally, the ratios of the different phospholipid species varied. The results relied on thin-layer chromatography and subsequent GC analysis to determine the identity of the different compounds.

A metabonomic analysis to unravel differences between 30 AS and 30 normozoospermic men using a GC-MS combination from Agilent (7890A and 5975C) identified 89 compounds in SP extracted with a solvent mixture of chloroform and methanol (2:1, *v*/*v*) [57]. According to the authors, these were amino acids, carbohydrates and fatty acids. Usually, the extraction with chloroform and methanol leads to a separation of polar and nonpolar species. Thus, this resulting mixture of polar and apolar molecules is astonishing. Benzoic acid, D-pinitol, cholecalciferol and the fatty acids C16:0, C18:1 and C19:0 were found to be more concentrated (by 1.15- to 1.38-fold), whereas Val was more than 14-fold less abundant in the SP of AS samples compared to controls. The increase in saturated and monounsaturated fatty acids might promote decreased sperm motility. Due to a study on fish that showed a positive effect of Val on the motility of European perch, the authors concluded that a decrease in Val might contribute to poor sperm motility in men.

In 2017, Gilany and colleagues investigated the polar components of SP samples of ten men with normal spermiograms, eleven with TESE-negative azoospermia and nine with TESE-positive azoospermia using untargeted GC-MS and chemometric methods [58]. They developed multivariate models to discriminate between the three groups. These authors identified discriminatory metabolites by applying different mathematical algorithms, such as correlation-optimized wrapping (to align the scan numbers of different samples in each section separately, minimizing the effect of retention time drift), evolving factor analysis and multivariate curve resolution–alternating least squares (to process the data and extract pure concentration profiles and mass spectra in each section). They identified five polar metabolites that are also described in the human metabolome database (HMDB), namely tartaric acid, 2,2,4,4,6,6-hexamethyl-1,3,5-trithiane (trithioacetone, a food additive as a flavoring agent), darwinol (a bicyclic monoterpene), 2-pyrrolidineacetate and 4,5-dimethoxy-1,2-benzenedicarboxylate (a metabolite of phthalate) as markers for patients with TESE-negative azoospermia. The occurrence of tataric acid in azoospermic men was explained by either a higher alcohol consumption or an impaired metabolism of tartaric acid by gastrointestinal bacteria. The authors further described 28 polar metabolites in the context of azoospermia that have not been mentioned so far in the HMDB.

The metabolome of sperm cells from AS patients (*n* = 30) without any obvious reason for this pathology, such as infections, varicocele, physical, chemical, immune, endocrine and chromosomal factors, abnormal karyotype or any systemic disease, was compared to that of normozoospermic donors (*n* = 30) in an untargeted approach by Zhao et al. [59]. Semen analysis was performed according to the fifth edition of the WHO guidelines [3]. Even though the authors stated that >4% of sperm of patients and donors were normomorphic, there are no data on sperm morphology in the respective table. Cells were separated from SP by density gradient centrifugation. Three samples were pooled. As an internal standard, 2-chlorophenylalanine was added. Cells were mixed with methanol–chloroform–water (4:1:1, by vol.) and lysed by ultrasound, and an aliquot of the supernatant was mixed with methoxyamine, N,O-bis(trimethylsilyl)trifluoroacetamide (BSTFA) and trimethylchlorosilane (TMCS) for derivatization in order to render the analytes sufficiently volatile. GC-MS using an Agilent 7890A-5975 GC mass spectrometer equipped with a nonpolar DB-5 capillary column (J&W Scientific, Folsom, CA, USA) revealed 33 components that were significantly different between the pathological and the control group. The concentrations of 6 compounds (benzoate, 2-deoxyerythriol, dithioerythriol, ethanolamine, orotate, zymosterol) were higher in patients, and 27 were found in lower concentrations, among them amino acids, organic acids and carbohydrates. For details, please see Table 4.

**Table 4:** Metabolites for pathological conditions in the context of male fertility identified by GC-MS. For the discriminatory metabolites, arrows indicate the direction of change. AS—asthenozoospermia, SP—seminal plasma, TESE—testicular sperm extraction.

Ref.	Pathological Condition/ Study Group 1	Controls/ Study Group 2	Sample	Discriminatory Metabolites
[48]	elevated abnormal cells (*n* = 16)	proven fathers (*n* = 8)	sperm	↓ PE ↑ PS, LPS
			SP	↑ PA, PS
			total semen	↓ fatty acids C18:0, C20:5, C22:6 ↑ fatty acids C16:0, C18:3, C24:0
[57]	AS (*n* = 30)	normozoospermic (*n* = 30)	SP	↓ Val ↑ benzoate ↑ cholecalciferol ↑ fatty acids C16:0, C18:1, C19:0 ↑ D-pinitol
[58]	nonobstructive azoospermia, TESE-negative (*n* = 11) TESE-positive (*n* = 9)	normozoospermic (*n* = 10)	SP	↑ darwinol ↑ 4,5-dimethoxy-1,2-benzenedicarboxylic acid (4,5-dimethoxyphthalate) ↑ 2,2,4,4,6,6-hexamethyl-1,3,5-trithiane ↑ 2-pyrrolidineacetate ((±)-homoproline) ↑ tartarate
[59]	AS (*n* = 10 pools of 3)	normozoospermic (*n* = 10 pools of 3)	sperm	↓ amino acids Cys, Glu, Leu, Trp ↓ amines 2-aminoethanethiol, 2-amino-1-phenylethanol, N-(3-aminopropyl)-morpholine, phenylethylamine ↓ 5-aminovalerate ↓ D,L-dihydrosphingosine ↓ glycerate ↓ *cis*-gondoate ↓ guanidinosuccinate ↓ lactate ↓ methylheptadecanoate ↓ methylmercaptopurine ↓ monoolein ↓ norvaline ↓ nucleosides guanosine, cytidine ↓ 3-phosphoglycerate ↓ phytosphingosine ↓ picolinate ↓ pipecolinate ↓ α-tocopherol ↓ *trans*-4-hydroxyproline ↑ benzoate ↑ 2-deoxyerythritol ↑ dithioerythritol ↑ ethanolamine ↑ orotate ↑ zymosterol

Analysis of SP samples of 80 idiopathic and 80 fertile Chinese men by GC-TOF MS on an Agilent 6890N gas chromatograph coupled to a Pegasus HT system revealed 333 metabolites that could be detected [60]. There were no differences in age, BMI or drinking and smoking status between the groups. Seventy of these compounds were validated by reference standards after application of quality control, of which (according to the respective table) 38 were differentially concentrated in patients vs. controls. Among these, 31 metabolites, namely N-acetyl glucosamine, cadaverine, citrate, dithiothreitol, ethanolamine, galactose, glycerate, hydroxyacetate, hypoxanthine, inositol, itaconate, pyroglutamate, rhamnose, threitol, threonate, uridine, xanthine, xylitol and the amino acids Ala, Asn, Asp, Glu, Gly, Ile, Phe, Pro, Ser, Thr, Trp and Val were found in lower levels. In contrast, gluconate, glucose, 4-hydroxyphenylacetate, mannose, spermidine, spermine, urea and Gln were found in higher levels in men with unexplained male infertility compared to healthy controls. Most of these metabolites correlated positively with sperm concentration, except glucose, mannose and urea, which correlated negatively. In contrast to that, glucose, mannose and urea correlated positively with the velocity of sperm, as determined by CASA, whereas all the others correlated negatively with the velocity of sperm. The authors concluded that the major metabolic signature of idiopathic infertility is an increased catabolism of some amino acids. It has to be mentioned that lipids and other apolar compounds were excluded from this study due to the extraction protocol. Furthermore, even though the study was published in 2017, only the “old” fourth edition of the WHO guidelines from 1999 [2] was used for sperm evaluation. Nevertheless, the investigation of samples from idiopathic patients by metabolomic approaches is absolutely necessary to understand the issues in these men where spermiogram parameters do not give any hint about subfertility. The metabolome of spermatozoa would probably give even more insights into idiopathic infertility.

## 3. Summary

LC-MS and ^1^H NMR are the most frequently used analytical methods for metabolomic studies investigating human semen. Because NMR is not as sensitive as mass spectrometric approaches, only abundant metabolites can be detected by NMR. Citrate, one of the abundant metabolites in human semen, with a concentration of about 30 mM (range ≈ 18–48 mM [61]) in the seminal fluid, mainly derives from the prostate. It has an impact on the semen pH value. Four NMR studies [19,20,21,23] and one LC study [42] reported lower citrate concentrations in patients with different semen pathologies (azoospermia, oligozoospermia, AS and OAT). However, in the SP of idiopathic subfertile patients, citrate was decreased compared to normozoospermic men, as shown by NMR and GC [21,59,60]. One NMR-based study reported an increased choline/citrate ratio in azoospermic patients [20], which could also be the result of a decreased citrate level. Due to this consensus, it is likely that a decrease in citrate really seems to be an issue in male infertility. However, there is one NMR study showing increased citrate levels in the SP of teratozoospermic men [30]. Lactate concentrations in SP are also high (≈15 mM [62]), and two NMR studies showed lower levels in the SP of azoospermic and OAT patients [19,27]. An increased choline/lactate ratio in azoospermic patients points to the same direction [20]. The reader should note that only a small number of indicative resonances (such as citrate and lactate) is normally considered in NMR studies. For sperm, GC analysis showed lower lactate concentrations in AS men [60] However, there was one NMR study and one LC study reporting higher lactate levels in terato- [30] and AS men [42]. Thus, the impact of lactate in subfertility needs further investigation. Two NMR studies and one LC study showed decreased GPC concentrations in the SP of azoospermic, oligozoospermic, low-semen-quality and idiopathic patients [9,19,21,41] but also after short-term sexual abstinence [9]. However, there was also one LC study reporting higher levels of GPC in low-quality semen [44].

Other metabolites that were detected by NMR, LC and GC and whose levels were reported to be different in more than one subfertile condition were the amino acids Ala, Arg, Glu, Gln, Leu, Lys, Phe, Pro, Tyr and Val as well as the biogenic amine spermine. Carnitines were detected by LC- and GC-MS and also differed in the semen of normozoospermic vs. subfertile men. The concentration changes were not always consistent for the same pathological condition. Thus, more studies are needed on these metabolites to verify their potential as biomarkers for distinct subfertile conditions.

## 4. Conclusions

At the moment, the HMDB contains more than 114,000 metabolites, and more than 351,000 reference spectra that have been experimentally acquired and computationally predicted by NMR, MS/MS and GC–MS [63]. When we look at the number of the first HMDB in 2007, which consisted of 2180 human metabolites, this reflects a more than 50-fold increase in known molecules that regulate functions within the human body.

Due to its momentary character, the metabolome represents a phenotype much better than the transcriptome. However, there is one considerable problem found in many studies. Most of the time, control groups are men attending the fertility clinic showing a normal spermiogram and, thus, should be diagnosed as idiopathic patients rather than healthy controls. Even though these men might be difficult to recruit, healthy controls need to be men of a defined age group with a normal spermiogram according to WHO guidelines who have already initiated pregnancy and fathered a child. As spermiogram disorders, such as AS, AT, OAT, OA, teratozoospermia and azoospermia, are easy to assess under a microscope, the idiopathic patients represent a big challenge for andrological centers. Thus, metabolomics could help to unravel the issues leading to male infertility with normal spermiogram parameters. Of course, parameters of lifestyle choices such as smoking and drinking habits need to be considered as factors that might influence fertility.

**Figure 1:**
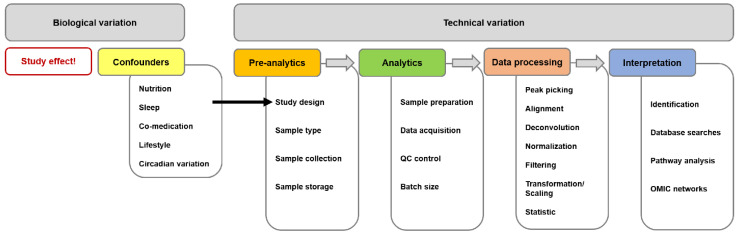
Sources for variations/for differing results in untargeted metabolomics experiments divided into biological and technical influences. Biological confounders can/should be controlled by a thorough study design. Each step of a common untargeted metabolome workflow (pre-analytics, analytics, data processing and interpretation) should be as stringent as possible and thoroughly documented. This figure and the legend were taken from the open access article [65] distributed under the terms of the Creative Commons CC BY license.

Contradictions between reports using the same analytical method could originate from various sources of variation (Figure 1). First, the classification of semen samples needs to be consistent with current WHO guidelines. Due to the highly dynamic nature of the metabolome, a variety of biological influence factors of the donors could have an effect and therefore have to be documented and—if possible—considered. Besides biological and/or environmental parameters such as age, BMI, nutrition, (co-)medication, lifestyle, dietary habits, circadian rhythm and many others, pre-analytical steps in study design, sample collection and handling are also among the most decisive ones for producing reliable analytical results [64]. Different workups lead to different moieties of metabolites and, thus, are not fully comparable.

Comprehensive control of all the parameters might be impossible, but a framework of standardization parameters has to be defined. Sufficient standardization parameters as described for other specimens such as serum, urine or cerebrospinal fluid have to be defined to facilitate proper sample collection, handling and storage [66,67,68].

Furthermore, technical aspects regarding different analytical techniques with different sample preparation strategies need to be validated and have to at least be supported by a proper number of replicates and have pooled internal quality controls of used sample materials as well as ideally commercially available external quality controls to enable valid data acquisition [69,70]. Additionally, the processing of raw data with bioinformatics tools including, e.g., alignment, peak picking, normalization, deconvolution, filtering and statistical analysis, has to be described transparently to allow a potential replication by other groups and to create the groundwork for multicenter studies and more harmonized data [71,72,73,74].

Finally, the interpretation of results originating from global untargeted metabolomics studies and the elucidation of underlying pathways and networks often relies on the quality of the identification of potential discriminating candidates. Thereby, analytical expertise and carefulness as well as the final verification of potential targets using reference standards is essential to avoid false-positive identifications and misinterpretation.

Just like that initiated for lipidomics of a certain body fluid, there is an urgent need for a worldwide metabolomics study on semen coordinated by a handful of scientists defining the criteria and the analytical methods as well as collecting the results afterwards. Only such a large-scale pre-defined study could really help in finding biomarkers for the distinct semen abnormalities. It also seems necessary that analytical chemists and biochemists take a joint look at the obtained data, as it would be highly desirable to verify the meaning of the achieved data.

## Figures and Tables

**Table 1:** Methods that have been used for semen metabolomics. The characteristics, advantages and disadvantages of spectroscopic and chromatographic methods are displayed.

Technique	Characteristics	Advantages	Disadvantages
**Spectroscopic Methods**			
Proton nuclear magnetic resonance (^1^H NMR)	Analytes are differentiated by the characteristic chemical shifts of dedicated functional groups; Assignment of ambiguous signals can be performed without further sample preparation (although time-consuming).	Nondestructive; Minimal sample handling; Quantitative data analysis is possible as signal intensity correlates directly with the analyte concentration; Established protocols for small and large analytes available.	Crowded spectra in mixture analysis; Expensive equipment and high maintenance costs; Insensitive.
Infrared (IR)	Light in the infrared range (ca. 900 nm) excites molecular vibrations; Particularly suitable for the excitation of polar groups such as -OH or C=O.	Inexpensive equipment; Characteristic wavelengths of stretching and bending vibrations represent a “fingerprint” of chemical compounds.	Mixtures are difficult to analyze; Monitors only the presence and absence of (polar) functional groups; Requires suppression of the intense water band in aqueous samples.
Raman	Excites vibrations of functional groups; Dependent on the reduced mass, the required energy is different; Compared to IR, more suitable for apolar residues.	Nondestructive; Can be performed in aqueous systems because the water vibration is excited only to a minor extent.	Mixtures are difficult to analyze; Monitors only the presence and absence of characteristic vibrations of functional groups; Risk of misinterpretation.
**Chromatography coupled to mass spectrometry (MS)**			
High-performance liquid chromatography (HPLC) MS	Most commonly used approach in “metabolomics” studies; Analytes are retained with different efficiencies depending on the affinity to the solvent or the stationary phase; Structure elucidation of unknown compounds (MS/MS).	Many well-established protocols available; Reversed-phase (RP) separation is more common than normal-phase (NP); Separation of nearly all compounds; High throughput is possible.	Requires relatively large amounts of solvent; Memory effects may occur; If gradients are used, great care is needed if ESI MS is used as the detection method since ESI is sensitive to solvent changes; Impact of the solvents on MS performance.
Gas chromatography(GC) MS	Only applicable to volatile compounds; Derivatization is often needed in order to enhance the analyte volatilities; Since MS detects ions in the gas phase, GC is often combined with MS detection.	Method of choice to determine many important compounds such as fatty acids; Excellent resolving power; Many protocols available.	Requires volatile compounds and/or derivatization to enhance volatility; Laborious and time-consuming.

## Data Availability

Not applicable.

## Abbreviations

α-AAA—α-aminoadipic acid, AS—asthenozoospermia, AT—asthenoteratozoospermia, ATR—attenuated total reflection, BMI—body mass index, CASA—computer-assisted semen analysis, CL—cardiolipin, DHET — dihydroxyeicosatrienoic acid, DSS—dimethyl-silapentane-sulfonate, EET—epoxyeicosatrienoic acid, ESI—electrospray ionization, FSH—follicle-stimulating hormone, FT—Fourier transformed, HPLC—high-performance liquid chromatography, GC—gas chromatography, GM3—monosialodihexosylganglioside, GPC—glycerylphosphorylcholine, GPE—glycerylphosphorylethanolamie, HETE—hydroxyeicosatetraenoic acid, HMDB—human metabolome database, IR—infrared, IS—internal standard, ICSI—intracytoplasmic sperm injection, IVF—in vitro fertilization, LC—liquid chromatography, LDL—low-density lipoprotein, LH—luteinizing hormone, LPC—lysophosphatidylcholine, LPI—lysophosphatidylinositol, LPO—lipid peroxides, MS—mass spectrometry, MS/MS—tandem mass spectrometry, NMR—nuclear magnetic resonance, NP—normal phase, OA—oligoasthenozoospermia, OAT—oligoasthenoteratozoospermia, PC—phosphatidylcholine, PCA—principal component analysis, PCR—polymerase chain reaction, PE—phosphatidylethanolamine, PG—phosphatidylglycerol, PGD (PGE, PGF)—prostaglandin D (E, F), PLS-DA—partial least-square discriminant analysis, PUFA—polyunsaturated fatty acid, QTOF—quadrupole time-of-flight, QTRAP—quadrupole ion trap, RP—reversed phase, SP—seminal plasma, TAG—triacylglycerol, TESE—testicular sperm extraction, TMS—tetramethylsilane, TSP—sodium trimethylsilyl propionate, UPLC—ultra-performance liquid chromatography, URSA—unexplained recurrent spontaneous abortion, VIP—variable importance in the projection, VLDL—very low density lipoprotein, WHO—World Health Organization.
